# CD133 and Ki-67 expression is associated with gastrointestinal stromal tumor prognosis

**DOI:** 10.3892/ol.2013.1564

**Published:** 2013-09-05

**Authors:** CANRONG LU, LICHENG LIU, XIN WU, WENTONG XU

**Affiliations:** Department of General Surgery, Chinese People’s Liberation Army General Hospital, Beijing 100853, P.R. China

**Keywords:** gastrointestinal stromal tumors, CD133, CD117, Ki-67, prognosis

## Abstract

CD133^+^ tumor cells have a greater potential ability for tumorigenesis, proliferation, invasion and metastasis compared with CD133^−^ tumor cells. Ki-67 is associated with cell proliferation in various tumors and has a markedly positive correlation with the prognosis of patients. However, there are a limited number of studies that have investigated the association between the prognosis of gastrointestinal stromal tumors (GISTs) and the two markers. The present study aimed to investigate CD133 and Ki-67 expression in GISTs and to explore their clinicopathological significance in the prognosis of patients with GISTs. A total of 111 GIST patients from the Chinese People’s Liberation Army (PLA) General Hospital were retrospectively followed up and immunohistochemistry was used to detect CD133, Ki-67 and CD117 expression in the tumor samples. The survival rates of the patients were analyzed using the Kaplan-Meier method. The log-rank test, χ^2^ test and Cox’s proportional hazards model were used to determine the association between CD133, Ki-67, CD117 expression and the prognosis of GIST. The 1-, 3- and 5-year survival rates were 93.0, 89.0 and 82.0%, respectively, in all the patients. However, in the patients with CD133^+^ or Ki-67^+^, the 1-, 3- and 5-year survival rates were 81.0, 61.5 and 50.0% and 83.0, 66.6 and 53.0%, respectively. Compared with the negative groups, the survival rates in the positive groups were statistically lower (CD133 log-rank, P=0.028; Ki-67 log-rank, P=0.002). The multivariate Cox analysis revealed that CD133 and Ki-67 expression were considerable factors in the prognosis of GIST patients (CD117, P=0.495; CD133, P=0.036; Ki-67, P=0.003). In conclusion, the positive expression of CD133 and Ki-67 was associated with a poor prognosis of GIST.

## Introduction

Gastrointestinal stromal tumors (GISTs) are mesenchymal tumors that arise from the gastrointestinal tract, showing differentiation toward the interstitial cells of Cajal and accounting for <1% of all gastrointestinal neoplasms (1). GISTs predominantly and positively express DOG-1 (98%) and CD117 (95%) immunoglobulin (Ig). The estimated incidence of GISTs is 10–20 million people annually worldwide. The majority of GISTs arise in the stomach (60%), small bowel (30%) and the esophagus and rectum (10%) (2), and the remaining are extragastrointestinal, comprising a wide spectrum from a curable disorder to a highly malignant disease. With regard to the molecular markers, previous studies have revealed that p53, CD147, MCT1, DDX39 and NKp30 are associated with the prognosis of GISTs (3–7). However due to the weakness in their correlation, these markers, which are different from tumor size, mitotic rate or tumor site, are rarely mentioned in predicting the recurrence risk of GISTs and the prognosis of patients.

CD133 is a novel plasma membrane glycoprotein that was first identified in humans as a hematopoietic stem cell marker (8) and is currently used for the differentiation of stem cells from several tissues and cancer types (9). In nude mice, CD133^+^ tumor cells have been identified to have a greater potential ability for tumorigenesis compared with that of CD133^−^ tumor cells (10,11). Other studies have indicated that CD133^+^ tumor cells have a greater potential ability for proliferation, invasion and metastasis compared with that of CD133^−^ cells (12–14). A study by Arne *et al*(15) revealed that CD133 was predominantly expressed in gastric GIST with KIT exon 11 mutations using the tissue microarray (TMA) method. This was defined as being a subgroup with a poor prognosis. There are no reports discussing the predictive value of the prognosis of GIST patients from a clinicopathological aspect.

Ki-67, as a nuclear marker, is closely associated with tumor cell proliferation. Ki67 has been identified to have a positive correlation with the prognosis of various malignant tumors, including GIST. One study has indicated that Ki-67 is a strong prognosticator, though it is less valuable than the mitotic rate in GIST (16). The study by Nakamura *et al*(17) supports the hypothesis that Ki-67 and the risk grade are useful for predicting the aggressive biological behavior of GIST.

The present study aimed to reveal the association between the molecular markers, CD133 and Ki-67, and the prognosis of GISTs. As the diagnosis index, CD117 has been found to be located at the tumor cell membrane and cytoplasm (18) and the positive rate recorded as high as 95% in GISTs. The predictive value of CD117 in the prognosis of GISTs was also explored.

## Materials and methods

### Study population and follow-up

A total of 111 patients with GIST were admitted to the Chinese People’s Liberation Army (PLA) General Hospital (Bejing, China) and underwent surgery between January 2004 and December 2010. The patients were retrospectively followed up. The clinical follow-up was completed in February 2011. The inclusion criteria consisted of an age of ≥18 years old, GISTs diagnosed by histopathological and immunohistochemical methods and receipt of no other previous treatment. The exclusion criteria consisted of female patients who were pregnant or lactating, patients who had developed other malignancies during past five years and patients with other serious diseases.

### Pathological examination of tumor samples

Paraffin wax sections (5-μm thick) of the GIST specimens were dewaxed in xylene and transferred to alcohol. The antibodies that were used were CD117 (rabbit anti-human polyclonal antibody; 1:100; Abcam, Cambridge, UK), Ki67 (rabbit polyclonal to proliferation marker; 1:1,000; Abcam) and CD133 (rabbit anti-human polyclonal antibody; 1:100; Biocare Medical, Concord, CA, USA). The endogenous peroxidase activity was blocked using 0.5% hydrogen peroxide in methanol and the sections were boiled in 10 mmol/l citrate buffer (pH 6.0) in a microwave oven for 150 sec for antigen retrieval. Non-specific binding was blocked by incubating the sections with 3% normal horse serum for 20 min. The sections were incubated overnight at 4°C with a 1:1,000 dilution mouse monoclonal antibodies for CD133, CD117 and Ki-67, respectively. Poly-peroxidase-anti-mouse/rabbit IgG was applied to the sections for 30 min at 37°C, then detected using 3,3′-diaminobenzine (DAB; Bioss, Beijing, China). The immunohistochemical reaction was developed with freshly prepared reagents of hematoxylin and mounted with glue (19,20). The immunohistochemical reactions were then visualized under high power magnification (x400) using an Olympus BH2 microscope (field width, 0.5 mm; Olympus Optical Co., Ltd., Tokyo, Japan) and scored into the following two categories based on the percentage of positively stained cells: CD117- and CD133-negative, <10% and -positive, >10%; and Ki-67-negative, <5% and -positive, >5%.

### Study ethics

Approval for this study was obtained from the Chinese PLA General Hospital Ethics Committee. Informed consent was obtained from the patients for the use of the clinical material for research purposes.

### Statistical analysis

SPSS 17.0 (SPSS Inc., Chicago, IL, USA) was used for the statistical analysis. The analysis was performed assuming a non-parametric distribution using the χ^2^ test. The actuarial survival rates were evaluated using Kaplan-Meier and log-rank tests. The multivariate survival analysis was performed using Cox’s proportional hazards model. All the tests were two-tailed and P<0.05 was considered to indicated a statistically significant difference.

## Results

### Clinical characteristics

A total of 111 GIST patients (59 male and 52 female) with a median age of 57 years were included in this study. Of these, 27 were incidental cases. The median follow-up time was 22 months (range, 3–80 months) (Table I).

### CD133, Ki-67 and CD117 expression in the tumor samples

**Figure 1:**
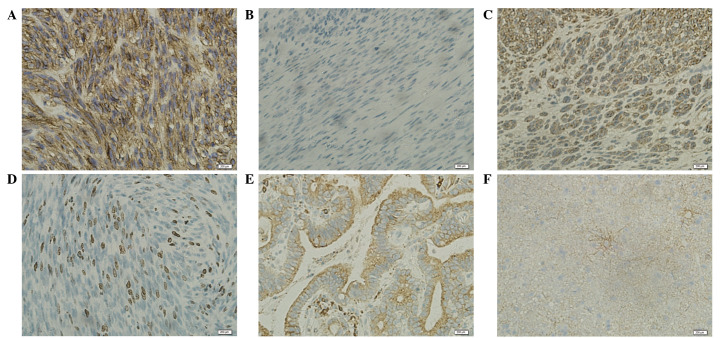
Immunohistochemistry of CD133, CD117 and Ki67 proteins in GIST and the controls. (A) CD133^+^ GIST. (B) CD133^−^ GIST. (C) CD117^+^ GIST. (D) Ki67^+^ GIST. (E) CD133^+^ gastric cancer. (F) CD133^+^ brain glioma cells. GIST, gastrointestinal stromal tumor. Bars, 200 μm.

The immunohistochemical results revealed that the positive expression rates for CD117, CD133 and Ki-67 were 86.5% (96/111), 43.2% (48/111) and 47.7% (53/111) in all the patients, respectively. The CD133 protein was expressed in the cell membrane or cell plasma, with a single focal expression (Fig. 1A). The Ki-67 protein was expressed in the nuclei of the GIST cells (Fig. 1B). The CD117 protein was expressed in the cell membrane or plasma (Fig. 1C). In the positive control group, there was no CD133 expression in the GIST cells (Fig. 1D), while CD133 was positively expressed in the gastric cancer and brain glioma cells (Fig. 1E and F). The histopathological type (spindle cell, epithelioid or mixed) was noted and the mitoses were counted using a 40X objective for 50 high-power fields (HPF), as recommended previously (21).

### Association between CD133, KI-67 and CD177 expression and clinicopathological characteristics of GISTs

**Figure 2:**
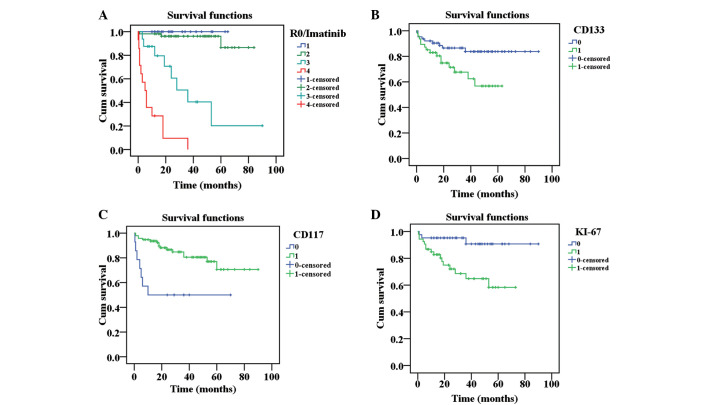
Analysis of the survival rates (Kaplan-Meier) and the comparison of the survival rates in the gastrointestinal stromal tumors (GISTs) groups (log-rank test). The highest survival rate was identified in patients that underwent a complete tumor resection and were administered imatinib (400 mg/day) post-operatively. The patients that did not undergo complete tumor resections and were not treated with imatinib post-operatively experienced the lowest survival rate (log-rank, P=0.000). (A) 1, Resected completely with imatinib; 2, resected completely without imatinib; 3, resected incompletely with imatinib; and 4, resected incompletely without imatinib. The survival rates in the (B) CD133^+^ or (D) Ki-67^+^ groups were statistically lower than those in the respective negative groups (CD133 log-rank, P=0.028; Ki-67 log-rank, P=0.002). (C) The survival rate was higher in the CD117^+^ group rather than the negative group (log-rank P=0.001). (B–D) 1, positive; 0, negative.

The survival analysis for all the GIST patients revealed that the 1-, 3- and 5-year survival rates were 93.0, 89.0 and 82.0%, respectively. The recurrence rate was 10.6% with a recurrence time of 6–20 months. The highest survival rate was identified in the patients who underwent a complete tumor resection and were administered imatinib (400 mg/day) post-operatively. However, in the patients who did not undergo complete tumor resections and were not treated with imatinib post-operatively, the survival rate was the lowest. Considering the molecular markers, the survival rates in the CD133^+^ or Ki-67^+^ groups were statistically lower than those in the negative groups (CD133, log-rank P=0.028; and Ki-67, log-rank P=0.002). However, the rate was higher in the CD117^+^ group compared with that of the CD117^−^ group (log-rank P=0.001) (Fig. 2).

According to the risk grade of the USA National Institute of Health (NIH) (21), the GIST cases in the present study consisted of five extremely low-risk, 15 low-risk, 16 medium-risk and 75 high-risk cases. The comparison using the parameters of tumor diameter, tumor site, mitotic rate, NIH risk and depth of invasion revealed statistically significant differences between the Ki-67^+^ and Ki67^−^ groups, though no difference was identified for the CD117 marker. Significant differences were identified between the CD133^+^ and CD133^−^ groups only when they were compared using the tumor diameter, mitotic rate and NIH risk. In the CD133^+^ group, the cases with a high NIH risk accounted for 83.3% (40/48). Within the remaining pathological characteristics, the CD34−, smooth muscle actin (SMA)-, desmin- and vimentin-positive rates were 79.4, 46.8, 15 and 84.8%, respectively (Table II).

The multivariate Cox model analysis suggested that CD133 and Ki-67 expression, along with the tumor site, tumor diameter, mitotic rate, invading depth, completion of the resection, intraoperative rupture and adjuvant therapy were significant prognosis predictive factors (P<0.05). However, age, gender, margin distance, mucosal erosion, biopsy and CD34 and CD117 expression were not considered significant prognosis predictive factors (P>0.05) (Table III).

## Discussion

The present study provided evidence that tumor size, mitotic index, tumor location and intraoperative tumor rupture are associated with the prognosis and recurrence of GISTs (22–24). Although p53, CD147, MCT1, DDX39 and NKp30 have been identified to be associated with the prognosis of GIST, the factors have never been considered prognostic predictors due to the weakness of their correlation.

CD133, also known as prominin-1 or AC133, is a novel plasma membrane glycoprotein that is composed of 865 amino acids with a molecular weight of 120 kDa. CD133 consists of an extracellular NH_2_-terminal, two extracellular ring structures, two small cysteine-rich intracellular cyclic structures and an intracellular COOH-terminal. The gene is located on 4p15.32 (8,25). The CD133 protein localizes to membrane protrusions and is often expressed on adult stem cells, where it is believed to function in maintaining stem cell properties by suppressing differentiation. Studies have shown that CD133 is expressed in neurogenic tumors (26) and in the stem cells of lung, pancreatic, liver, prostate, gastric and colorectal cancer. In one previous study, CD133^+^ tumor cells were identified to have a greater potential ability for tumorigenesis compared with that of the CD133^−^ tumor cells (10,11). Other studies have indicated that CD133^+^ tumor cells had a greater potential ability for proliferation, invasion and metastasis than CD133^−^ cells (12–14). In addition, studies (27,28) have indicated that CD133 is highly expressed in a variety of malignancies and was often observed to be associated with a poor prognosis. Furthermore, using the TMA method, Arne *et al*(15) revealed that CD133 was predominantly expressed in gastric GISTs with KIT exon 11 mutations, which was known as a subgroup with a poor prognosis. According to the previous findings, the positive expression of CD133 may be closely associated with survival. In the present study, the close follow-up of 111 GIST patients revealed that the 1-, 3- and 5-year survival rates of the CD133^+^ group were lower than those of the CD133^−^ group. The present study further indicated that the expression of CD133 was a considerable factor in predicting the prognosis of GIST, as well as the tumor size, tumor location, mitotic index, depth of invasion and NIH risk classification. The findings coincided with the results of the study by Arne *et al*(15), in which a univariate survival analysis demonstrated a significant correlation between the presence of the CD133 protein and a shorter overall survival (hazard ratio, 2.23; P=0.027). The multivariate analysis revealed that CD133 provided additional information on patient survival compared with age, gender, NIH risk classification and mutational status. Based upon the comprehensive recognition that CD133^+^ tumor cells have the characteristics of cancer stem cells, CD133 may play a significant role in the occurrence and development of GIST. Combining previous results and those of the present study, a new therapeutic approach targeting CD133 and a practical application using CD133 in predicting the prognosis for GIST may be a promising new approach, however, further studies are necessary.

The Ki-67 protein, also known as MKI67, exists in actively proliferating cells in the G_1_, S and G_2_ phases, and is a proliferation-related nuclear marker of tumor cells (29). Certain studies have demonstrated that Ki-67^+^ expression is closely associated with the aggressive biological behavior of tumor cells in GISTs (17). The marker represents a good prognostic predictor for GISTs (30). However, the significance of Ki-67 in predicting the prognosis of GISTs remains in dispute.

Wong *et al*(16) identified that Ki-67 was less reliable than the mitotic count, although it proved to be useful in assessing the proliferation rate of the tumor cells in GISTs. The prognostic predictive value of Ki-67 in GISTs may have been evaluated more objectively in a large size sample case survival study with the various prognostic factors being taken into account. This was one of the aims of the present study. The study identified that the 1-, 3- and 5-year survival rates of the Ki-67^+^ GIST group were lower than those of the Ki-67^−^ group. The survival analysis further indicated that Ki-67 expression was also a significant prognostic predictor for GISTs. The Wald index of Ki-67 and the mitotic rate were similar (8.868 vs. 11.446), which indicated that Ki-67 was another useful molecular marker in predicting the prognosis of GISTs.

With regard to the molecular markers, CD117^−^ expression was believed to be associated with an early post-operative recurrence in GISTs (31). This was confirmed in the present study. The expression of CD117 was not associated with the prognosis of GISTs. In summary, the present study indicated that the positive expression of CD133 and Ki-67 were indicators of poor prognosis in GISTs. However, the smaller sample volume was a limitation in the present study. The study may promote the clinical application of the two markers and provide insight into novel therapeutic targets in the treatment of GISTs in the future.

## Figures and Tables

**Table I:** Clinical characteristics of patients with GISTs.

Variable	Value
Gender, n	
Male:female	59:52
Age, years	
Median (range)	57 (18–82)
Presentation, n	
Symptomatic	84
Incidental	27
Follow-up time, months	
Median (range)	22 (3–80)

GISTs, gastrointestinal stromal tumors.

**Table II:** Pathological parameters of GISTs in CD117, CD133 and Ki-67 proteins.

Variable1	Total, n	CD117, n		P-value	CD133, n		P-value	Ki-67, n		P-value
		
		+	−		+	−		+	−	
Diameter										
<5 cm	26	23	3	0.993	6	20	0.032^b^	6	20	0.004^a^
≥5 cm	85	73	12		42	43		47	38	
Site										
Stomach	45	40	5		18	27		14	31	
Small bowel	34	31	3	0.250	15	19	0.829	17	17	0.005^a^
Others	31	25	7		15	17		22	10	
Mitotic rate										
≥5 MF/50 HPFs	61	52	9	0.886	30	31	0.000^b^	38	23	0.001^a^
<5 MF/50 HPFs	50	44	6		18	32		15	35	
NIH risk										
Extremely low	5	4	1		1	4		2	3	
Low	15	13	2	0.978	2	13	0.014^b^	4	11	0.008^a^
Middle	16	14	2		5	11		3	13	
High	75	65	10		40	35		44	31	
Depth of invasion										
Mucosa	15	14	1		5	10		6	9	
Muscular	17	13	4		8	9		3	14	
Serous	72	63	9	0.546	33	39	0.680	38	34	0.010^a^
Adjacent tissue	7	6	1		2	5		6	1	

a The differences were statistically significant between the Ki-67^+^ and Ki-67^−^ groups. There was no difference between the CD117 groups.

b Significant differences were observed between the CD133^+^ and CD133^−^ groups only when compared by the tumor diameter, mitotic rate and NIH risk.

GISTs, gastrointestinal stromal tumors; MF, mitotic figures; HPFs, high power fields; NIH, National Institute of Health.

**Table III:** Multivariate survival analysis using Cox’s proportional hazards model in GISTs.

						95% CI for HR	
						
Variable	B	Wald	df	P-value	Hazard rate	Lower	Upper
Age (>60 vs. ≤60)	0.023	1.696	1	0.193	1.024	0.988	1.060
Gender (male vs. female)	−0.234	0.346	1	0.556	0.791	0.363	1.726
Margin distance (>5 vs. ≤5 cm)	0.269	1.381	1	0.240	1.344	0.821	2.201
Mucosal erosion (yes vs. no)	1.404	1.864	1	0.172	4.073	0.542	30.582
Biopsy (no vs. yes)	−0.303	0.502	1	0.429	0.739	0.319	1.708
CD34 (positive, >10%; negative, <10%)	−0.027	0.101	1	0.922	0.937	0.536	1.680
CD117 (positive, >10%; negative, <10%)	−0.214	0.466	1	0.495	0.808	0.437	1.491
Site^a^	0.622	25.015	1	0.000	1.863	1.460	2.378
Diameter (>5 vs. ≤5 cm)	0.132	32.876	1	0.000	1.141	1.091	1.194
CD133 (positive, >10%; negative, <10%)	0.221	2.094	1	0.036	1.250	1.014	1.534
Ki-67 (positive, >5%; negative, <5%)	1.887	8.868	1	0.003	6.602	1.906	22.863
Mitotic rate (>5/HPF vs. ≤5/HPF)	1.195	11.446	1	0.001	3.303	1.653	6.589
Depth of invasion^b^	1.205	7.539	1	0.006	3.336	1.412	7.883
Complete resection (no vs. yes)	2.807	24.674	1	0.000	16.555	5.470	50.104
Intraoperative rupture (no vs. yes)	−1.899	12.562	1	0.000	0.150	0.052	0.428
Adjuvant therapy (no vs. yes)	1.757	35.579	1	0.000	5.796	3.254	10.325

P<0.05.

a site (extra-gastrointestinal vs. gastrointestinal).

b Depth of invasion (mucosa, submucosa, muscular and serosa; unfavourable parameter, serosa vs. favourable parameter, mucosa).

GISTs, gastrointestinal stromal tumors; CI, confidence interval; HR, hazard ratio; HPF, high power field; B, regression coefficient; df, degree of freedom.
